# Experience with patient-specific guides, instead of general surgical experience, improves the accuracy of 3D-guided corrective osteotomies

**DOI:** 10.1007/s00068-026-03179-4

**Published:** 2026-04-21

**Authors:** M. L. Buist, A. M. L. Meesters, J. W. Colaris, J. N. Doornberg, K. ten Duis, S. J. C. Tabernée Heijtmeijer, P. C. Jutte, P. A. J. Pijpker, N. W. L. Schep, A. D. Smelt, H. C. van der Veen, A. J. H. Vochteloo, F. F. A. IJpma, N. Assink

**Affiliations:** 1 3D lab, University Medical Center Groningen, University of Groningen, Groningen, 9713 GZ The Netherlands; 2 Department of Trauma Surgery, University Medical Center Groningen, University of Groningen, Groningen, 9713 GZ The Netherlands; 3 Department of Orthopaedic Surgery, University Medical Center Groningen, University of Groningen, Groningen, 9713 GZ The Netherlands; 4 Department of Orthopaedics and Sports Medicine, Erasmus University Medical Center, Rotterdam, 3015 GD The Netherlands; 5 Department of Orthopedic Trauma, Flinders University, Flinders Medical Centre, Adelaide, 5042 Australia; 6 Department of Oral and Maxillofacial Surgery, University Medical Center Groningen, University of Groningen, Groningen, 9713 GZ The Netherlands; 7 Department of Trauma Surgery, Maasstad Ziekenhuis, Rotterdam, 3079 DZ The Netherlands; 8 Centre for Orthopeadic Surgery OCON, Hengelo, 7555 DL The Netherlands

**Keywords:** Patient-specific guides, Corrective osteotomy, 3D-guided osteotomy, 3D virtual surgical planning, 3D printing, Limb reconstruction

## Abstract

**Purpose:**

This study investigated whether the accuracy of 3D-guided osteotomies is influenced by surgeon experience. Two types of experience were assessed: (1) general surgical experience, defined as self-reported years in practice as an orthopaedic trauma surgeon, and (2) prior use of patient-specific guides (PSGs). Secondary aim was to evaluate whether accuracy varies by anatomical location.

**Methods:**

24 orthopaedic-trauma surgeons performed 75 corrective osteotomies on cadaveric long-bones (43 single-cut, 32 double-cut; 107 cuts in total). Each osteotomy was preoperatively planned on CT-derived 3D-reconstructions, and PSGs were manufactured to guide the cuts. Postoperative CT-scans were used to compare planned and executed osteotomy planes. Surgeons completed a questionnaire reporting years of practice and annual PSG usage. A Spearman’s rank-order correlation was used to test differences in accuracy versus experience, a Kruskal-Wallis test was performed to explore differences across anatomical locations.

**Results:**

General surgical experience ranged from 0 to 25 years (median 9), and PSG-usage from 0 to 20 cases annually (median 2). No association was found between general surgical experience and osteotomy accuracy (*p* = 0.406 and *p* = 0.548). In contrast, PSG-experience correlated with improved accuracy for angular and translational deviation of the osteotomy plane (*p* = 0.027 and *p* = 0.029). In addition, midshaft osteotomies of the lower extremity showed smaller angular deviations but larger translational errors compared with metaphyseal (*p* < 0.001 and *p* = 0.003).

**Conclusion:**

Experience with PSGs, rather than general surgical experience, improves accuracy in 3D-guided osteotomies, highlighting the need for training and repeated use. In contrast to metaphyseal, midshaft lower-extremity osteotomies are executed less accurately because fewer anatomical reference points are available.

**Supplementary Information:**

The online version contains supplementary material available at 10.1007/s00068-026-03179-4.

## Introduction

Malunions can occur in up to 17% of patients, depending on the fracture location and treatment method [1, 2]. Posttraumatic malunions can result in pain, function loss, instability, and an increased risk of osteoarthritis [3]. Corrective osteotomy surgery can restore alignment and joint function in cases of a symptomatic or severe malunion, by accurately cutting and realigning the affected bone. Conventionally, this procedure is performed using 2D imaging and intraoperative visual assessment to guide free-hand bone correction. However, due to the multiplanar nature of these deformities, the procedure can be technically demanding and less accurate, often leading to unpredictable outcomes [4, 5]. In recent years, the use of 3D virtual surgical planning and 3D-printed patient-specific surgical guides (PSGs) has grown substantially, particularly for 3D-guided corrective osteotomy surgery [6–9]. These 3D virtual surgical plans and PSGs are designed to facilitate complex three-dimensional corrections, aiming to improve osteotomy accuracy and streamline surgical procedures.

Most studies reporting on the use of PSGs to guide corrective osteotomy surgery only report on accuracy based on postoperative 2D radiographs despite the multiplanar correction [10]. Postoperative 3D evaluation of these corrections is not common in research or clinical practice. Yet, PSGs are designed to simplify corrective osteotomy procedures; therefore, it is often assumed that any orthopaedic trauma surgeon can implement the PSGs without requiring additional training. However, several studies have indicated that the clinical implementation of 3D technology involves a learning curve [11, 12]. Whether this also applies to 3D-guided corrective osteotomy surgery is not yet known or demonstrated.

The primary aim of this study was to determine whether the surgeon’s experience influences the accuracy of 3D-guided osteotomies in the long-bones of the extremities. We asked ourselves: (1) In corrective osteotomy surgery using patient-specific 3D-printed surgical guides, does surgery performed by experienced surgeons, compared with less experienced surgeons, result in higher surgical accuracy?; (2) In 3D-assisted corrective osteotomy surgery, do corrections at varying anatomical locations result in different levels of surgical accuracy?

## Methods

### Study design

In June 2025, the University Medical Center Groningen (Groningen, The Netherlands), hosted the first AO Masters Course on 3D-guided Osteotomies for Posttraumatic Deformities. A total of 75 corrective osteotomies of upper and lower extremities were planned using 3D technologies and performed on Thiel embalmed human cadavers, utilizing 3D-printed PSGs. These procedures formed the practical foundation for the educational program. 24 orthopaedic trauma surgeons from various countries participated. Each participant completed a questionnaire regarding their general surgical experience and prior use of PSGs.

During the course, participants were divided into random pairs, and each pair performed around six corrective osteotomies with 3D-printed patient-specific surgical guides. After completion of the osteotomies, postoperative CT-scans were made to evaluate the accuracy of the osteotomy planes as described in the following sections.

The primary outcome of this study was to compare the accuracy of the performed osteotomies to the two categories of experience of the surgeons:

1. General Surgical Experience (hereafter: “General Experience”). Defined as the self-reported years of practice as orthopaedic trauma surgeon.
2. Prior use of Patient-Specific Guides (hereafter: “PSG Experience”). Defined as the number of times per year that the surgeon uses 3D printed surgical guides.

The secondary outcome of this study was whether the accuracy varied by anatomical location of the applied PSG.

### 3D virtual surgical planning

**Fig. 1:**
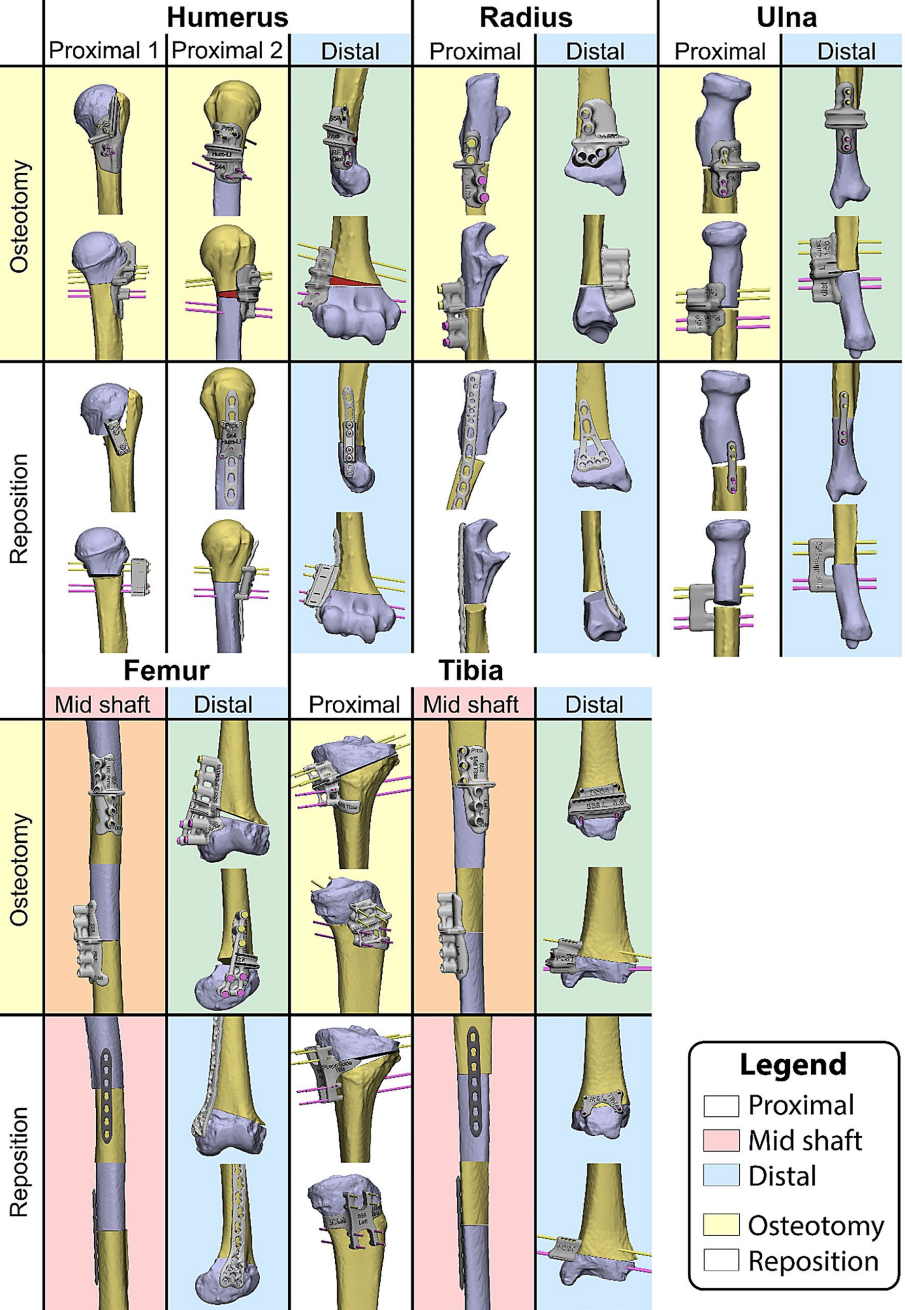
Examples of planned osteotomies in each of the anatomical locations. The top row shows the osteotomy guides, the bottom row displays the reposition, with either a repositioning guide or an osteosynthesis plate. The repositioning is for illustration only, repositioning was not analysed in this study

Prior to the course, four Thiel-embalmed human anatomical specimens underwent a full-body CT-scan (slice thickness: 0.6 mm). For each specimen, bilateral corrective osteotomies were planned at the following anatomical locations: proximal humerus, distal humerus, proximal radius, distal radius, proximal ulna, distal ulna, mid-shaft femur, distal femur, proximal tibia, mid-shaft tibia, and distal tibia, resulting in 75 planned corrections. Examples of corrections in each of the anatomical locations are displayed in Fig. 1.

**Fig. 2:**
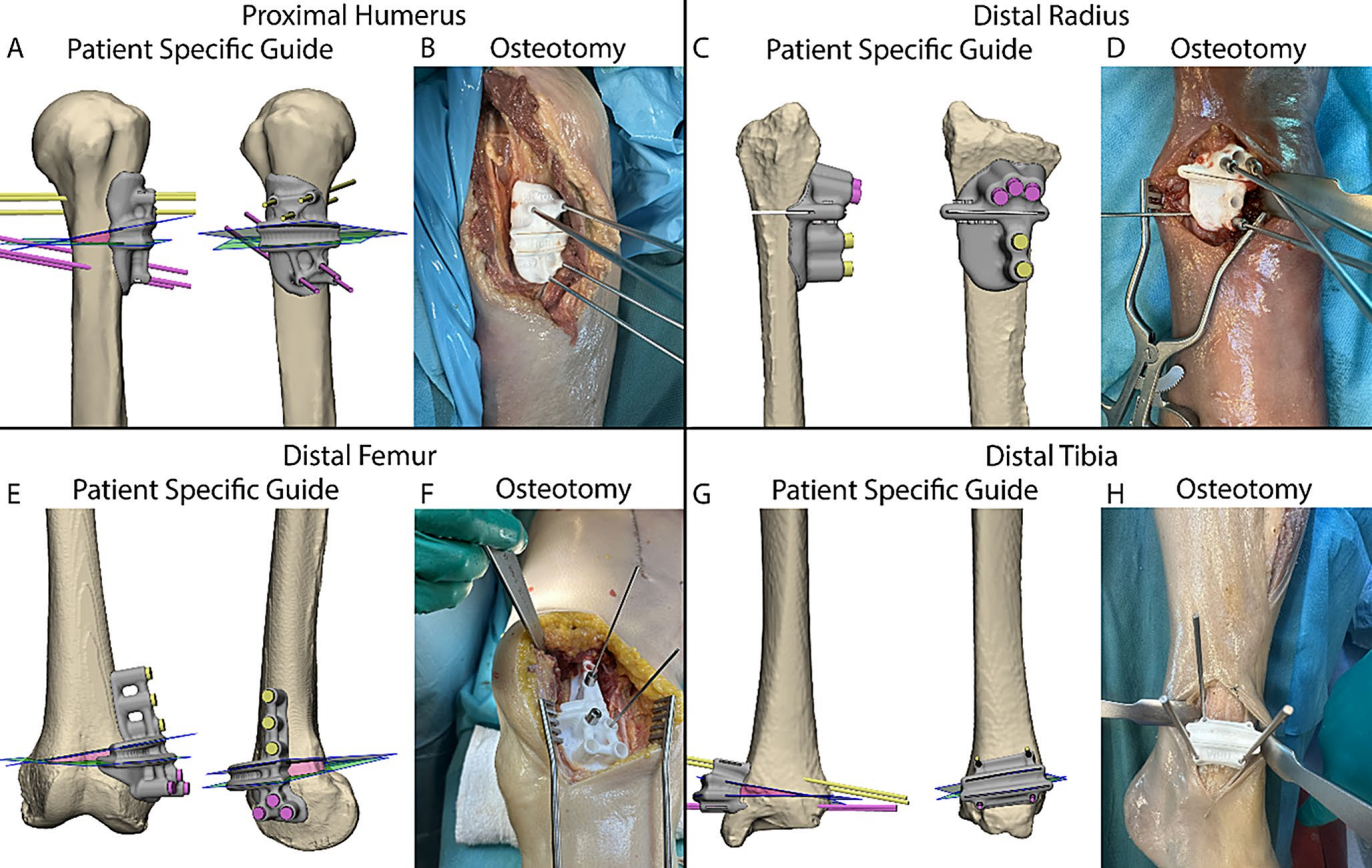
Four examples of Patient-Specific Guides during the procedure. **A** and **B** show the virtual surgical plan and patient-specific guide placement in the bodies for a Proximal Humerus, **C** and **D** for a Distal Radius, **E** and **F** for a Distal Femur, and **G** and H for a Distal Tibia. In **A **and **G **the proximal and distal k-wires are dislayed in yellow and purple, respectively, and in **C **and **E **the planned screw trajectories are displayed in yellow and purple, the planned osteotomy planes are displayed in green

Preoperative segmentation of the CT-scans was performed using Mimics software (version 27.0; Materialise, Belgium) to create 3D models of all long bones, similar to the methods described by Assink et al. [13]. Segmentation was based on a preset bone threshold (Hounsfield Units ≥ 226). Virtual osteotomy planes were then defined in the 3-Matic software (version 19.0; Materialise, Belgium) as often accounted in clinical practice. The virtual surgical plan was created by experienced technical physicians in consultation with senior surgeons, based on illustrative historical patient cases. Depending on the correction type, one or two planes per anatomical site were planned: single cut osteotomies for open wedge or rotational corrections, and double cut for closed wedge or shortening procedures. Figure 2 illustrates various types of corrections that were used during the course, with two examples of corrections with a single cut osteotomy and two examples of corrections with a double cut. All cadaveric bones exhibited normal anatomy; therefore, corrective plans intentionally introduced non-anatomical positions to simulate deformity correction. PSGs were subsequently designed by experienced technical physicians and guide design specialists in 3-Matic, tailored to the planned osteotomy planes and bone morphology. For the PSGs two commonly used correction techniques, the pre-drilling technique and the K-wire technique were used. The only difference in these techniques is the method to achieve repositioning of the bone, which is either with screws or k-wires (see Supplementary Materials 1) [14].

### Procedure

**Fig. 3:**
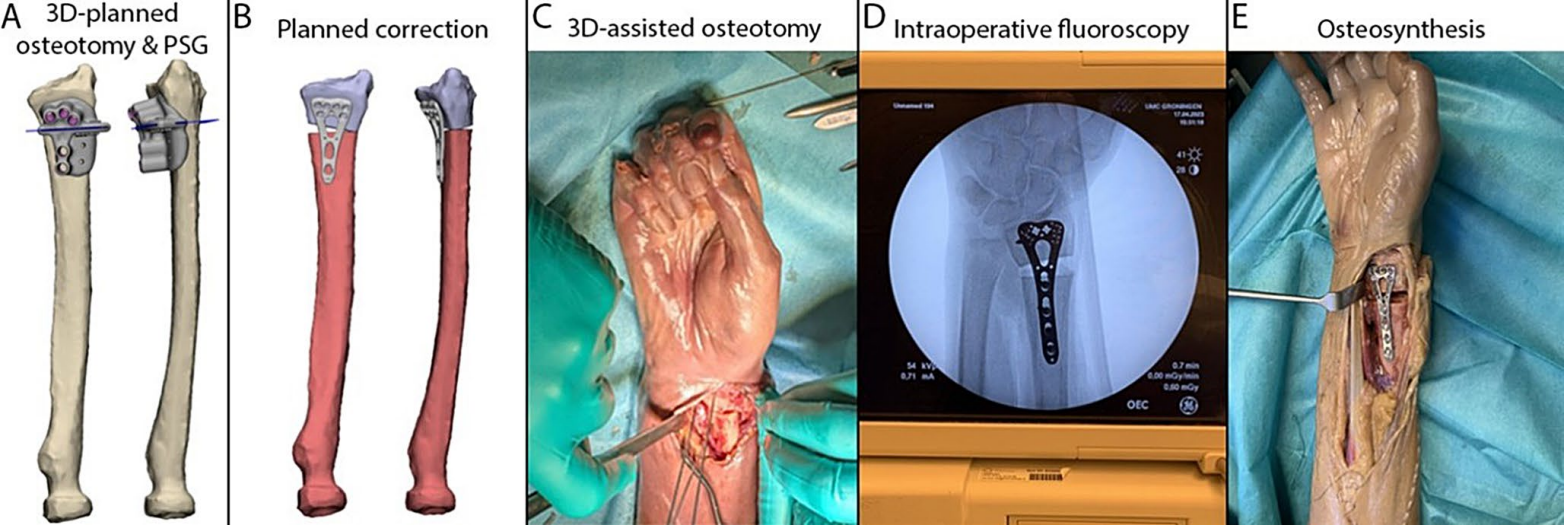
Steps of the procedure. Virtual surgical plan and Patient-Specific Guide (PSG) design (**A**) and planned correction (**B**). Execution of the planned osteotomy (**C**) with intraoperative fluoroscopy (**D**), and the end result with plate fixation (**E**)

Each procedure began with the surgical approach as deemed fit by the orthopaedic surgeon for the required exposure for the planned osteotomy. The PSG was positioned after exposing the bone. Four examples of the PSG placement are depicted in Fig. 2. The PSG was then fixed to the bone using 2.0 mm K-wires, after which the osteotomy was executed through the PSG with a saw blade. In case of the pre-drilling technique, the PSG was also used to drill the screw holes for the plate. Surgeons were allowed to use fluoroscopy for intraoperative verification; the use of fluoroscopy was left to the surgeons’ discretion. All steps of the procedure are illustrated in Fig. 3.

### Postoperative analysis

**Fig. 4:**
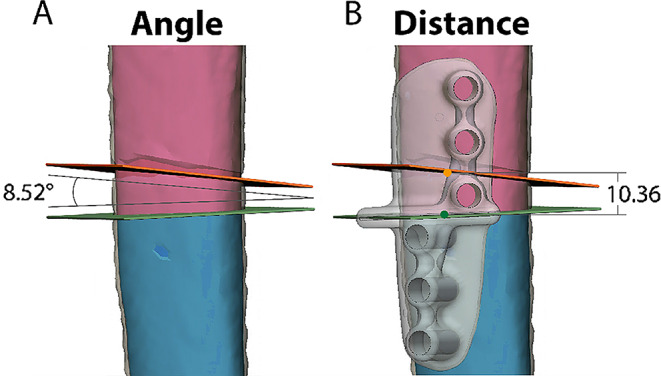
Example of angle and distance measurements of the osteotomy plane. Pink and Blue: the two bone fragments of the planned resection. Green: planned resection plane and entry point. Light Gray, see-through, the Patient-Specific osteotomy guide (PSG) in the planned position. Dark gray, see-through, the postoperative bone projected on top of the plan. Orange: postoperative location of the plane and entry point. In **A** the angle between the green (planned) and orange (executed) planes is measured, in this case 8.52 degrees. In **B** the distance between the green (planned) and orange (executed) entry point dots is measured, in this example 10.36 mm

After the procedure, the osteosynthesis materials were removed immediately due to course regulations. Therefore, only osteotomy cuts could be analysed rather than the full procedure including repositioning. The postoperative CT-scans were segmented using the same protocol as the preoperative segmentation. The 3D models were imported in the 3-Matic software, and the postoperative bone fragments were aligned to their corresponding preoperative models. Subsequently, a plane was fitted to the osteotomy surface of each fragment. For all osteotomies, angular and translational deviations were calculated using an automated protocol. The measurements were performed by one observer experienced in 3D virtual surgical planning. The two metrics were calculated in the following manner:

1. Angular deviation of the osteotomy plane (Angle, degrees): Difference in orientation between the two planes, see Fig. 4a.
2. Translational deviation (Distance, millimetres): Difference between the planes’ entry points, defined as the intersection with the original bone surface closest to the guide’s centre of gravity, see Fig. 4b.

### Statistical analysis

The data was non-normally distributed, therefore non-parametric tests were used. For the primary outcome, Spearman’s rank-order correlation was used to assess relationships between both accuracy metrics (Angle and Distance) of the osteotomy plane and both surgeon experience variables (“General Experience” and “PSG Experience”). Since the surgeons worked in pairs, the General Experience and PSG Experience values were averaged across both surgeons.

For the secondary outcome, a Kruskal-Wallis test was performed to explore differences in osteotomy accuracy (both Angle and Distance) across all anatomical locations. In the Kruskal-Wallis test the 11 anatomical locations were grouped and compared to the two accuracy metrics (Angle and Distance) as well as the two surgeon experience variables (“General Experience” and “PSG Experience”). The latter was done to verify that there were no variations in experience per anatomical location. No post-hoc pairwise comparison of the 11 groups was performed. Instead, a Mann–Whitney U test compared the osteotomy accuracy variables between two groups of anatomical locations in the lower extremities:

1. Group 1: Mid-shaft osteotomies (tibia and femur)
2. Group 2: Metaphyseal osteotomies (tibia and femur)

To assess the reliability of the angle and distance measurements, a second observer repeated all measurements for one of the four cadavers (*n* = 32 osteotomy cuts). Inter-observer agreement was evaluated using a two-way mixed-effects intraclass correlation coefficient (ICC), with the absolute-agreement definition and single-measure ICC. Reliability was calculated separately for the angular (degrees) and translational (millimetres) deviations.

All analyses were performed using SPSS (version 30.0; IBM Corp., Armonk, NY). Statistical significance was set at *p* < 0.05, using two-tailed tests.

## Results

The participating surgeons reported surgical experience ranging from 0 to 25 years (median 9), and patient-specific guide (PSG) usage ranged from 0 to 20 clinical cases per year (median 2). The 75 performed corrections consisted of 43 single cut osteotomies and 32 double cut osteotomies, resulting in a total of 107 executed osteotomy cuts.

Across all cuts, the median deviation between planned and achieved osteotomy position was 5.5° (IQR 3.2–8.3°) for angular deviation and 2.8 mm (IQR 0.8–7.9 mm) for translational deviation. Surgeons with greater general surgical experience (upper half) demonstrated median deviations of 5.7° and 3.1 mm, compared with 5.2° and 2.7 mm for those with less experience (lower half). In contrast, surgeons with higher PSG usage showed median deviations of 4.3° and 2.3 mm, while those with lower PSG usage had a median of 6.5° and 3.8 mm. Implying that the more PSG experienced half of the surgeons got 33% closer to the planned cutting plane in angle and and 40% closer to the translational deviation, than the less PSG experienced half.

### Primary outcome: correlation between osteotomy accuracy and experience

#### General experience

**Fig. 5:**
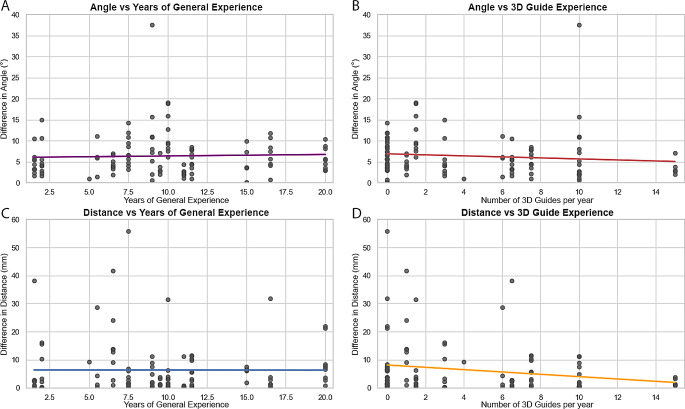
Scatter plots and regression lines showing accuracy of the osteotomy plane in terms of Angle (top) and Distance (bottom) deviations in relation years of general surgical experience (left), and annual patient-specific guide use (right). In both graphs on the left (**A** and **C**) the trend line is more-or-less a flat line. So, there is no association between the accuracy and years of surgical experience. In both graphs on the right (**B** and **D**) a decreasing trend is visible, indicating a higher accuracy when the annual PSG use increases

There was no association between general surgical experience and osteotomy accuracy in terms of Angle and Distance (*p* = 0.406 and *p* = 0.548, respectively), see the top part of Table 1, and Fig. 5a and c. The osteotomy does not get more accurate when a surgeon has more general surgical experience.

#### PSG experience

Experience with PSGs demonstrated a negative correlation with both accuracy metrics, Angle and Distance (*p* = 0.027 and *p* = 0.029, respectively), see the bottom part of Table 1, and Fig. 5b and d. This indicates that the accuracy improves when the number of performed 3D-guided cases per year increases.

**Table 1:** Spearman’s rank-order correlation coefficient, 95% confidence interval and p-values of the Spearman’s rank-order correlation from the difference in angle and distance between planned and postoperative osteotomy planes, correlated to the years of surgical experience and the number of Patient-Specific Guides (PSGs) used per year

	General Experience		PSG Experience	
	Correlation Coefficient (95% CI)	*P*-value (2-tailed)	Correlation Coefficient (95% CI)	*P*-value (2-tailed)
*Angle (°)*	0.081 (-0.116–0.272)	**0.406**	-0.213 (-0.392 – -0.019)	**0.027**
*Distance (mm)*	0.059 (-0.138–0.251)	**0.548**	-0.211 (-0.391 – -0.017)	**0.029**

### Secondary outcome: anatomical location

**Fig. 6:**
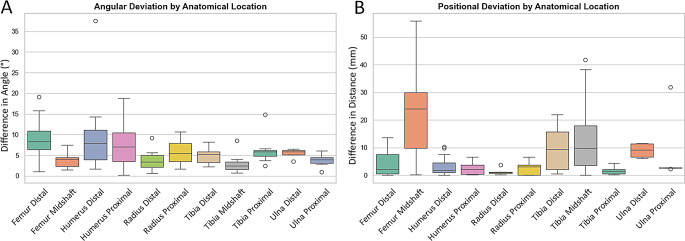
Boxplots showing difference in Angle (°) and Distance (mm) of the osteotomy planes across anatomical locations. The differences between the anatomical locations are statistically significant, with *p* = 0.007 for the Angular Deviation and *p* < 0.001 for the Positional Deviation. Two notable outliers are visible: a 38° rotational deviation at the distal humerus, likely caused by inverted guide placement, and a 32 mm translational deviation at the proximal ulna, an early case performed by surgeons without prior PSG experience. These outliers illustrate how early-stage handling errors can substantially increase variability, particularly in anatomically challenging or PSG-naïve situations. Besides that, a large range of values is visible in the positional deviation of the midshaft osteotomies, these do not have large outliers but a large range throughout the whole population

The boxplots presented in Fig. 6 provide a detailed overview of the variability in osteotomy accuracy across different anatomical regions. As shown, both the angular deviation (Fig. 6a) and the distance deviation (Fig. 6b) demonstrate region-specific differences (*p* = 0.007 for Angle; *p* < 0.001 for Distance), indicating that the accuracy of the osteotomy planes depended on the body region in which the procedure was performed. Additionally, surgeon-related factors did not differ across locations. There were no significant differences in overall surgeon experience or use of PSGs between the 11 anatomical locations (*p* = 0.997 and *p* = 0.994, respectively). This confirms that the observed accuracy differences were not driven by variations in surgeon experience.

To test our hypothesis on the performance of midshaft corrections compared to other lower extremity corrections, we compared the midshaft osteotomies (tibia and femur) to metaphyseal corrections in the same bones. This showed a significantly lower deviation in angle in the midshaft osteotomies (*p* < 0.001) and a significantly larger distance in the midshaft (*p* = 0.003), see Table 2. The midshaft group had distance values ranging from 0 to 56 mm, without large outliers, while in the metaphyseal group the largest distance was only 22 mm.

**Table 2:** Median, IQR, and p-value of the Mann-Whitney U test, for the lower extremity osteotomies, divided in the midshaft and metaphyseal groups

	Midshaft group Median (IQR)	Metaphyseal group Median (IQR)	*P*-value (2-tailed)
Angle (°)	3.1 (1.8–4.4)	6.1 (4.4–8.3)	< 0.001
*Distance (mm)*	11.3 (7.2–33.1)	2.6 (0.6–8.1)	**0.003**

### Inter-observer reliability

Inter‑observer reliability for the postoperative measurements was good for the angular deviation (ICC = 0.74), and excellent for the translational deviation (ICC = 0.997). Both ICC values were statistically significant (*p* < 0.001). These results indicate that the measurement protocol yields consistent and reproducible angular and translational deviation values.

## Discussion

In recent years, 3D-printed patient-specific surgical guides have been increasingly adopted in orthopaedic trauma surgery, particularly for corrective osteotomies. This study aimed to assess whether surgeon experience and anatomical position influence the ability to use these guides effectively and achieve accurate osteotomies. The results from studying 107 osteotomy cuts, performed by 24 surgeons, revealed that proper application of these surgical guides involves a learning curve, even among highly experienced surgeons. Furthermore, extra care should be taken with midshaft osteotomies, as these are considerably more challenging to position the PSG due to less anatomical reference points and therefore less accurate than metaphyseal osteotomies. These findings underscore that there is a learning curve when using PSGs, demonstrating that accuracy improves with dedicated practice and guide-specific experience.

Although PSGs are designed to simplify complex multiplanar corrective osteotomies, our results suggest that their effective use still involves a learning curve. Surgeons who performed more 3D-guided cases per year achieved higher accuracy, while no relationship was found with general surgical experience. However, it should be emphasized that all participating surgeons were trained orthopaedic trauma specialists, ensuring a baseline level of surgical proficiency. This indicates that, while PSGs are a valuable tool for simplifying corrective osteotomies, their effectiveness depends on the intraoperative handling more so than overall surgical experience of orthopaedic trauma surgeons. This is in line with the findings of Beek et al. and Zhu et al. [11, 15], who demonstrated a learning curve in the use of PSGs in both 3D-assisted orthognathic and reconstructive jaw surgery. Beek et al. observed that the learning curve continued to improve even after five years of PSG use. Our findings are also consistent with the findings of Jacquet et al., who reported a similar learning curve for PSGs in high-tibial osteotomies [16]. Our study found an average deviation of 5.45° and 2.76 mm between the planned and achieved osteotomy cuts, regarding the accuracy of the osteotomy cuts. This was largely in line with other studies reporting the accuracy of PSG-guided osteotomies in long bones. For example, a clinical study on distal radius osteotomies showed placement errors of 2.4 mm and 3.8° using patient-specific guides [17], while a cadaveric study on the tibia and femur demonstrated displacement errors of approximately 0.7–0.8 mm with angular deviations of 2.0° and 5.2°, respectively [18]. Our study included a few outliers that increased the overall average deviation. For example, in one case a surgeon pair positioned the cutting guide upside down, resulting in a substantial angular error. This incident likely reflects limited prior experience with PSGs, further underscoring the influence of PSG-specific familiarity on osteotomy accuracy. Although osteotomy plane accuracy, our primary outcome, does not translate directly one-to-one into the final correction accuracy of malunited bones, achieving a precise osteotomy cut remains an essential prerequisite for optimal correction. For the distal radius, for example, deformities exceeding 2 mm in radial shortening or 10° in dorsal tilt have been associated with worse functional outcomes [19, 20]. In our study, surgeons demonstrated median deviations of 1.1 mm and 3.4° in the performed osteotomy plane of the distal radius, which was well below this threshold, indicating that the precision achieved in our cohort seems sufficient to support reliable anatomical realignment. Our study adds to the current literature by providing quantitative evidence that targeted experience with PSGs enhances performance, emphasizing that successful implementation of 3D-guided osteotomies requires not only surgical skill, but also dedicated practice with these 3D tools.

The values from the above-mentioned clinical studies are largely based on proximal and distal corrections, such as High Tibial Osteotomies. These types of osteotomies generally achieve a high accuracy. However, mid-shaft osteotomies are generally deemed to be more difficult for the PSG placement [21]. This may be explained by the uniform cylindrical shape of the diaphysis, which provides fewer unique anatomical landmarks for guide positioning, making the guide less constrained to a single location. In line with previous studies, we saw in our study that osteotomy accuracy also varied by anatomical location. Midshaft femoral and tibial osteotomies showed significantly larger errors in distance compared to proximal or distal locations in the lower extremities, while their angular accuracy was higher. On the contrary, the planned cutting planes in the midshaft regions were all planned perpendicular to the bone axis, which makes it easier to achieve higher angular precision. To ensure appropriate positioning of the PSGs in the mid-shaft region, extra care is warranted in future cases. In clinical practice, the distance between the surgical guide and the proximal and/or distal joint is typically known and can be verified to some extend with fluoroscopy and a measuring tape. Other solutions are to use Augmented Reality or to optimize the PSG design itself [22, 23]. For example, Reyniers et al. demonstrated that providing an additional sterile, patient-specific 3D-printed bone model together with the PSG helped the surgeons to verify and adjust the guide’s position intraoperatively by matching its contours directly to the bone model. With this method, they were able to reduce the risk of misplacement in the mid-shaft region [23].

This study has several limitations. First, all procedures were performed on Thiel-embalmed human anatomical specimens without malunions. The absence of callus formation may have made positioning of the PSGs more challenging than in clinical cases where deformity-related anatomy provides additional reference. However, with matured malunions, the callus formation is not always visible either. In addition, although Thiel-embalmed specimens replicate tissue handling well, they lack key intraoperative factors such as active bleeding and soft-tissue swelling, which in clinical surgery can further complicate guide positioning. As such, the setup in our study was slightly less demanding than in vivo conditions, and these differences may further amplify the disparity between surgeons with more versus less PSG experience. Besides that, the osteotomies were performed by surgeon pairs with varying experience. Therefore, measurements had to be averaged between the two surgeons, which may have reduced the ability to detect operator-dependent variability. Yet, this represents clinical practice where often a senior surgeon teaches the less experienced surgeon. Another limitation is that due to the setting of the study, the participating surgeons were less engaged in the guide designs. However, the guides were designed conform clinical practice and based on real cases. Lastly, only the accuracy of the osteotomy cut could be evaluated, accuracy of the postoperative alignment after fixation could not be assessed. This was due to the fact that the osteosynthesis hardware had to be removed by the end of the session before the postoperative CT-scans could be made (regulatory issue). A future prospective series is necessary to analyse the accuracy of the repositioning and fixation of the bone fragments, and most importantly assess its impact on patient outcome.

In conclusion, our study shows that experience with patient-specific guides, rather than general surgical experience, is associated with improved positioning of surgical guides and accuracy in performing 3D-guided osteotomies. This suggests that the surgical learning curve of osteotomies can be shortened by training with PSG’s. Midshaft osteotomies remain more challenging for translational accuracy due to the uniform bone shape, therefore care should be taken with midshaft osteotomies with no clearly visible anatomical reference points for guide positioning. These findings highlight that while patient-specific guides simplify multiplanar osteotomies, proper training and repeated use are important to achieve optimal results.

## Electronic supplementary material

Below is the link to the electronic supplementary material.

Supplementary Material 1

## Data Availability

The data that support the findings of this study are not openly available due to reasons of sensitivity and are available from the corresponding author upon reasonable request.

## References

[1] Katt B, Seigerman D, Lutsky K, Beredjiklian P. Distal radius malunion. J Hand Surg Am. 2020 May;45(5):433–42 10.1016/j.jhsa.2020.02.008. 10.1016/j.jhsa.2020.02.008 32220492

[2] Bushnell BD, Bynum DK. Malunion of the distal radius. J Am Acad Orthop Surg. 2007 Jan;15(1):27–40. 10.5435/00124635-200701000-00004. 10.5435/00124635-200701000-00004 17213380

[3] Patel I, Young J, Washington A, Vaidya R. Malunion of the tibia: a systematic review. Medicina* (B. Aires). *2022 Mar;58(3):389. 10.3390/medicina58030389. 10.3390/medicina58030389 PMC8956117 35334565

[4] D’Amelio A, Van Lieshout EMM, Wakker AM, Verhofstad MHJ, Van Vledder MG. 3D-printed patient specific instruments for corrective osteotomies of the lower extremity. Injury. 2022 Nov;53:S53–8. 10.1016/j.injury.2022.08.069. 10.1016/j.injury.2022.08.069 36075778

[5] Varaschin A, et al. Personalised high tibial osteotomy surgery is accurate: an assessment using 3D distance mapping. Appl Sci. 2024 Oct;14:9033. 10.3390/app14199033.

[6] Lal H, Patralekh MK. 3D printing and its applications in orthopaedic trauma: a technological marvel. J Clin Orthop Trauma. 2018 Jul;9(3):260–8. 10.1016/j.jcot.2018.07.022. 10.1016/j.jcot.2018.07.022 PMC6128305 30202159

[7] Tack P, Victor J, Gemmel P, Annemans L. 3D-printing techniques in a medical setting: a systematic literature review. Biomed Eng Online. 2016 Dec;15(1):115. 10.1186/s12938-016-0236-4. 10.1186/s12938-016-0236-4 PMC5073919 27769304

[8] Dasari SP, et al. Patient-specific instrumentation for medial opening wedge high tibial osteotomies in the management of medial compartment osteoarthritis yields high accuracy and low complication rates: a systematic review. J ISAKOS. 2023 Jun;8(3)163–176. 10.1016/j.jisako.2023.02.001. 10.1016/j.jisako.2023.02.001 36931505

[9] Kampkuiper N, et al. Clinical added value of 3D printed patient-specific guides in orthopedic surgery (excluding knee arthroplasty): a systematic review. Sci Bus Media Deutschland GmbH. Dec 01 2025 Springer. 10.1007/s00402-025-05775-2. 10.1007/s00402-025-05775-2 PMC11872977 40025308

[10] Aman ZS, DePhillipo NN, Peebles LA, Familiari F, LaPrade RF, Dekker TJ. Improved accuracy of coronal alignment can be attained using 3D-printed patient-specific instrumentation for knee osteotomies: a systematic review of level III and IV studies. Arthroscopy. 2022 Sep;38(9):2741–58. 10.1016/j.arthro.2022.02.023. 10.1016/j.arthro.2022.02.023 35247513

[11] Beek D-M, et al. A learning curve in 3D virtual surgical planned orthognathic surgery. Clin Oral Investig. 2023 Apr;27(7):3907–15. 10.1007/s00784-023-05013-2. 10.1007/s00784-023-05013-2 PMC10329591 37083986

[12] Balling H. Learning curve analysis of 3D-fluoroscopy image-guided pedicle screw insertions in lumbar single-level fusion procedures. Arch Orthop Trauma Surg. 2018 Nov;138(11):1501–9. 10.1007/s00402-018-2994-x. 10.1007/s00402-018-2994-x 29982886

[13] Assink N, et al. Development of patient-specific osteosynthesis including 3D-printed drilling guides for medial tibial plateau fracture surgery. Eur J Trauma Emerg Surg. 2024 Feb;50(1):11–9. 10.1007/s00068-023-02313-w. 10.1007/s00068-023-02313-w PMC10924019 37391531

[14] Oldhoff MGE, et al. 3D-assisted corrective osteotomies of the distal radius: a comparison of pre-contoured conventional implants versus patient-specific implants. Eur J Trauma Emerg Surg. 2024 Feb;50(1):37–47. 10.1007/s00068-023-02415-5 10.1007/s00068-023-02415-5 PMC10924012 38261077

[15] Zhu W, Choi WS, Wong MCM, Pu JJ, Yang W, Su Y. The learning curve of computer-assisted free flap jaw reconstruction surgery using 3D-printed patient-specific plates: a cumulative sum analysis. Front Oncol. 2021 Sep;11. 10.3389/fonc.2021.737769. 10.3389/fonc.2021.737769 PMC8481918 34604076

[16] Jacquet C et al. Patient-specific high-tibial osteotomy’s ‘cutting-guides’ decrease operating time and the number of fluoroscopic images taken after a brief learning curve. Knee Surg Sports Traumatol Arthrosc. 2020 Sep;28(9):2854–62. 10.1007/s00167-019-05637-6. 10.1007/s00167-019-05637-6 31352498

[17] Willemsen K, et al. 3D-printed saw guides for lower arm osteotomy, a comparison between a synthetic CT and CT-based workflow. 3D Print Med. 2021 Dec;7(1):13. 10.1186/s41205-021-00103-x. 10.1186/s41205-021-00103-x PMC8082893 33914209

[18] Kievit AJ, Dobbe JGG, Streekstra GJ, Blankevoort L, Schafroth MU. Predicted osteotomy planes are accurate when using patient-specific instrumentation for total knee arthroplasty in cadavers: a descriptive analysis. Knee Surg Sports Traumatol Arthrosc. 2018 Jun;26(6):1751–8. 10.1007/s00167-017-4721-5. 10.1007/s00167-017-4721-5 PMC5966490 28948339

[19] Lalone EA, Grewal R, King GJW, MacDermid JC. A structured review addressing the use of radiographic measures of alignment and the definition of acceptability in patients with distal radius fractures. 2015 Dec;10(4):619–29. 10.1007/s11552-015-9772-9. 10.1007/s11552-015-9772-9 PMC4641087 26568715

[20] Brogren E, Hofer M, Petranek M, Wagner P, Dahlin LB, Atroshi I. Relationship between distal radius fracture malunion and arm-related disability: a prospective population-based cohort study with 1-year follow-up. BMC Musculoskelet Disord. 2011;12. 10.1186/1471-2474-12-9. 10.1186/1471-2474-12-9 PMC3032765 21232088

[21] Oldhoff MGE, Alvarez CP, Ten Duis K, Doornberg JN, Assink N, IJpma FFA. Patient-specific implants combined with 3D-printed drilling guides for corrective osteotomies of multiplanar tibial and femoral shaft malunions leads to more accurate corrections. Eur J Trauma Emerg Surg. 2025 Dec;51(1):53. 10.1007/s00068-024-02755-w. 10.1007/s00068-024-02755-w PMC11761992 39856352

[22] Fernández-Fernández T, et al. Augmented reality-assisted placement of surgical guides and osteotomy execution for pelvic tumour resections: a pre-clinical feasibility study using 3D-printed models. Cancers (Basel). 2025 Jul;17(13):2260. 10.3390/cancers17132260. 10.3390/cancers17132260 PMC12249251 40647556

[23] Reyniers P, Verrewaere D, van Es E, Colaris J, Schweizer A, Verstreken F. The role of 3D technology in corrective osteotomy for forearm malunion. J Hand Surg Eur Vol. 2025 Jun;50(6):771–80. 10.1177/17531934251327286. 10.1177/17531934251327286 40145427

