# Publisher Correction: Experience with patient-specific guides, instead of general surgical experience, improves the accuracy of 3D-guided corrective osteotomies

**DOI:** 10.1007/s00068-026-03202-8

**Published:** 2026-04-30

**Authors:** M. L. Buist, A. M. L. Meesters, J. W. Colaris, J. N. Doornberg, K. ten Duis, S. J. C. Tabernée Heijtmeijer, P. C. Jutte, P. A. J. Pijpker, N. W. L. Schep, A. D. Smelt, H. C. van der Veen, A. J. H. Vochteloo, F. F. A. IJpma, N. Assink

**Affiliations:** 1 3D Lab, University Medical Center Groningen, University of Groningen, Groningen, 9713 GZ The Netherlands; 2 Department of Trauma Surgery, University Medical Center Groningen, University of Groningen, Groningen, 9713 GZ The Netherlands; 3 Department of Orthopaedic Surgery, University Medical Center Groningen, University of Groningen, Groningen, 9713 GZ The Netherlands; 4 Department of Orthopaedics and Sports Medicine, Erasmus University Medical Center, Rotterdam, 3015 GD The Netherlands; 5 Department of Orthopedic Trauma, Flinders University, Flinders Medical Centre, Adelaide, 5042 Australia; 6 Department of Oral and Maxillofacial Surgery, University Medical Center Groningen, University of Groningen, Groningen, 9713 GZ The Netherlands; 7 Department of Trauma Surgery, Maasstad Ziekenhuis, Rotterdam, 3079 DZ The Netherlands; 8 Centre for Orthopeadic Surgery OCON, Hengelo, 7555 DL The Netherlands

**Publisher Correction: European Journal of Trauma and Emergency Surgery (2026) 52:140**

10.1007/s00068-026-03179-4

In this article the title was incorrectly given as

‘Experience with patient-specific guides, insteatientad of general surgical experience, improves the accuracy of 3D-guided corrective osteotomies’

but should have been ‘Experience with patient-specific guides, instead of general surgical experience, improves the accuracy of 3D-guided corrective osteotomies’.

The Original Article has been corrected.

