# Supplementary material for: Experience with patient-specific guides, instead of general surgical experience, improves the accuracy of 3D-guided corrective osteotomies
Source: Eur J Trauma Emerg Surg. 2026 Apr 21;52(1):140. doi: 10.1007/s00068-026-03179-4 (PMC13099835; doi:10.1007/s00068-026-03179-4)
Supplement: Supplementary file 1 — Supplementary Material 1 [file 68_2026_3179_MOESM1_ESM.docx]

**Supplementary materials**


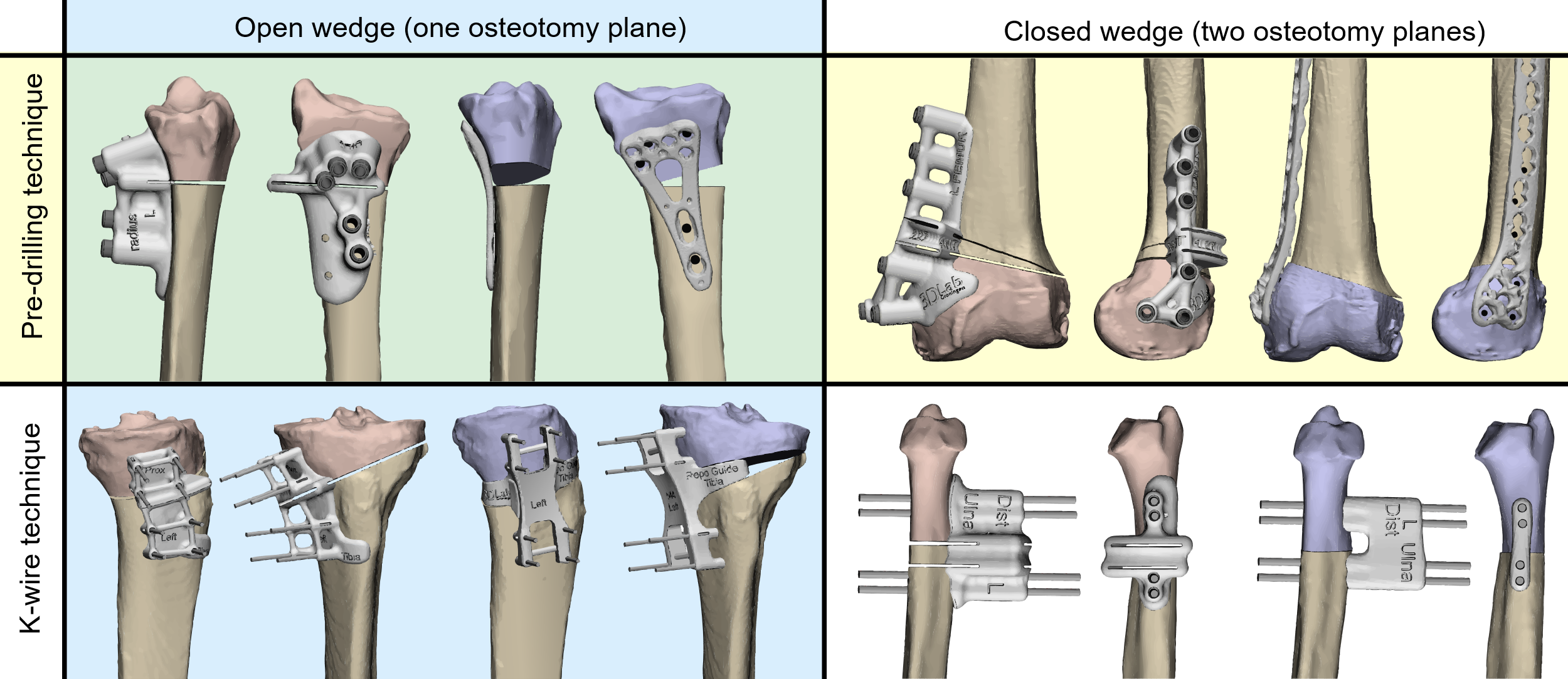


**Supplementary Figure 1** Type of corrections. Left column shows examples of open wedge corrections with one osteotomy plane. Right column illustrates examples of corrections with two osteotomy planes. The top row demonstrates the pre-drilling technique; the bottom row demonstrates the K-wire technique. With the pre-drilling technique predetermined screw holes for the fixation plate were drilled using a drill bit for which sleeves were designed in the PSG [23]. The plate then served as a repositioning tool, ensuring alignment through the pre-drilled holes. With the K-wire technique predetermined Kirschner wires (K-wires) were placed using guiding sleeves in the patient-specific guide (PSG), followed by a secondary PSG, the repositioning guide, which forced the K-wires in a planned direction to achieve the planned correction
